# Determining total energy expenditure in 3–6-year-old Japanese pre-school children using the doubly labeled water method

**DOI:** 10.1186/s40101-022-00301-4

**Published:** 2022-08-05

**Authors:** Keisuke Teramoto, Kodo Otoki, Erina Muramatsu, Chika Oya, Yui Kataoka, Shoji Igawa

**Affiliations:** 1 Aichi University of Education, Hirosawa 1, Igaya, Kariya, Aichi Japan; 2 Kyushu Kyoritsu University, Jiyugaoka 1-8, Yahata, Kitakyushu, Fukuoka Japan; 3 Humanitec Junior College, Minami Hamada 4-2, Yokkaichi, Mie Japan; 4 Kyushu Women’s Junior College, Jiyugaoka 1-1, Yahata Nishi, Kitakyushu, Fukuoka Japan; 5 Nippon Sport Science University, Kamoshida 1221-1, Yokohama, Kanagawa Japan

**Keywords:** Total energy expenditure, Doubly labeled water method, Pre-school Japanese children

## Abstract

The doubly labeled water (DLW, ^2^H_2_^18^O) method for calculating the total production of CO_2_ over several days is currently considered to be the most accurate technique for the measurement of total energy expenditure (TEE), and the results obtained using this method have been used to review energy requirements. Presently, there is limited data available on TEE in Japanese children. The objective of this study was to assess the TEE in pre-school Japanese children using the DLW method. We used a cross-sectional population of 140 children (69 boys and 71 girls) aged 3–6 years. TEE was measured using the DLW method over 8 days under free-living conditions. The average weights (kg) of the boys and girls were 15.6 ± 2.5 and 15.0 ± 2.1 for the 3–4 years old and 19.8 ± 3.8 and 19.6 ± 2.7 for the 5–6 years old, respectively. The corresponding TEE (kcal/day) was 1260.9 ± 357.8 and 1265.2 ± 408.0, and 1682.3 ± 489.0 and 1693.1 ± 473.3, respectively, showing a significant difference with respect to age. Furthermore, TEE per body weight (kcal/kg/day) was 83.2 ± 29.2 and 84.9 ± 26.6, and 85.4 ± 23.2 and 86.7 ± 22.6, respectively. However, when TEE was adjusted for body weight or fat-free mass, there were no age or sex differences. We conclude that in Japanese children, TEE in those aged 3–4 years was similar to the current Ministry of Health recommendations. However, TEE in children aged 5–6 years was slightly higher than the recommendations. Based on these findings, the present results obtained from a large number of participants will provide valuable reference data for Japanese children.

## Introduction

Childhood obesity is becoming a serious public health issue worldwide, and it is an important determinant of obesity risk [1, 2]. Worldwide, 40 million children are overweight and obesity around the world has seen an increase of 10 million since 2000 [3].

The societal factors that contribute to higher rates of obesity include extreme and rapid changes in lifestyle, physical activity, and diet that accompany urbanization and rapid economic development. In other words, increased television viewing and decreased exercise time have led to a static lifestyle; furthermore, high-fat food intake has increased [4]. Physically active children have lower adiposity, more favorable lipid profiles (triglycerides and high-density lipoprotein cholesterol level), and increased cognitive function compared to their inactive counterparts [5]. Energy deficiency due to poor nutrient intake may cause stunted growth in children and increase their susceptibility to disease. There are approximately 149 million children over the age of five suffering from stunting worldwide [6].

Therefore, assessment of total energy expenditure (TEE) is an essential component of clinical nutrition in children [6], and TEE data can provide information on energy requirements. This is based on the concept that daily energy intake should be equivalent to the total daily energy expenditure to maintain energy balance. The energy requirements of children are defined as the amount of food energy needed to balance TEE at a desirable level of physical activity and to support optimal growth and development [7, 8].

The doubly labeled water (DLW) method can measure TEE in a cumulative and non-invasive manner over a long period of time. It is considered to be the most accurate method of assessment because measuring TEE in an unrestricted activity environment (free-living environment) is an important factor when targeting children [6]. This method has the advantage of being easy to use in field studies, as the procedure requires only urine and/or saliva sampling following the administration of the isotopes. Therefore, it is possible to know the total energy expenditure by measuring it over several days of daily life. The DLW method has been shown to be able to calculate TEE with an accuracy of 1–3% and a precision of 2–8% of the estimated value [9]. Therefore, it is a very effective method for calculating the energy expenditure of young children who have difficulty limiting their activities for certain periods of time. Most of the data on TEE using the DLW method have been obtained from Caucasian children [7, 8] with limited data available for Asian and Japanese children probably because the costs associated with these measurements are high, and data collection and analyses is time-consuming [6, 10].

Most studies conducted worldwide have used the DLW technique to calculate TEE data with a small number of participants. As shown in previous studies [11], energy expenditure studies in large populations require data to be collected from various geographical regions and a diverse socioeconomic background with a large number of participants. For studies on children, it is essential to collect data in early childhood, which is particularly difficult. The purpose of the present study was to determine the reference values of TEE in 3- to 6-year-old pre-school Japanese children in a large population and compare them with those observed in previous studies.

## Methods

Healthy Japanese children were enrolled for the body composition reference study from kindergartens in Miyazaki and Iwate prefectures in Japan. Informed consent was provided by the parents before commencement of the measurements. The data were obtained from a cross-sectional sample of 140 participants (69 boys and 71 girls) aged 3–6 years. Twelve participants were excluded from the study because they were either absent for urine collection or could not fully ingest the DLW. Data for those who skipped the measurement or those with incomplete data were excluded from the analyses. All data were obtained between 2009 and 2017. Although the data in this study were measured over a long period of time, the Japanese Association for Human Auxology stated that “it is appropriate to continue to use the reference values calculated from the anthropometric data published by the Ministry of Health, Labour and Welfare and the Ministry of Education, Culture, Sports, Science and Technology in 2000 as standard values when evaluating the anthropometric status of Japanese children” [12]. Therefore, our study was based on the idea that the body size of Japanese children did not change significantly during this period. This study was conducted in compliance with the Declaration of Helsinki, the Ethical Guidelines for Clinical Research, and the Act on the Protection of Personal Information. This study received ethical approval from the research ethics committee of Aichi University of Education, Japan.

All measurements were performed in a room at approximately 23 °C, with the participants wearing thin undergarments. During the measurement of the TEE, weight was measured to the nearest 0.02 kg using a balance beam scale and height was measured to the nearest 0.1 cm. The body mass index (BMI) was calculated by dividing body weight (kg) by height squared (m^2^). Body composition was estimated using a 2-component model. Impedance was determined using a four-terminal impedance analyzer (TP-95 K, Toyo Physical, Japan). Each subject wore garments with no shoes or socks and laid supine on a bed with their limbs extended away from the trunk of their body. After cleaning all skin contact areas with alcohol, current electrodes (Red Dot-2330; 3 M Health Care, USA) were placed on the dorsal surfaces of the right hand and right foot at the distal metacarpals and metatarsals, respectively. Detector electrodes were also applied at the right pisiform prominences of the wrist and between the medial and lateral malleoli at the ankle. This bioelectrical impedance analyzer generated an excitation current of 500 mA at a single frequency of 50 kHz. Fat-free mass (FFM) was calculated using the equation of Kushner et al. [13] and the total body water (TBW) component (hydration) of FFM [14]. Fat mass (FM) was calculated as the difference between body weight and FFM. The FFM index (FFMI) and FM index (FMI) were calculated by dividing each of the BMI components, FFM and FM, by height^2^ (BMI = FFM, kg/height, m^2^ + FM, kg/height, m^2^). These two component indices are known as the FFMI and FMI, both of which are discrete and adjusted for body size.

**Fig. 1:**
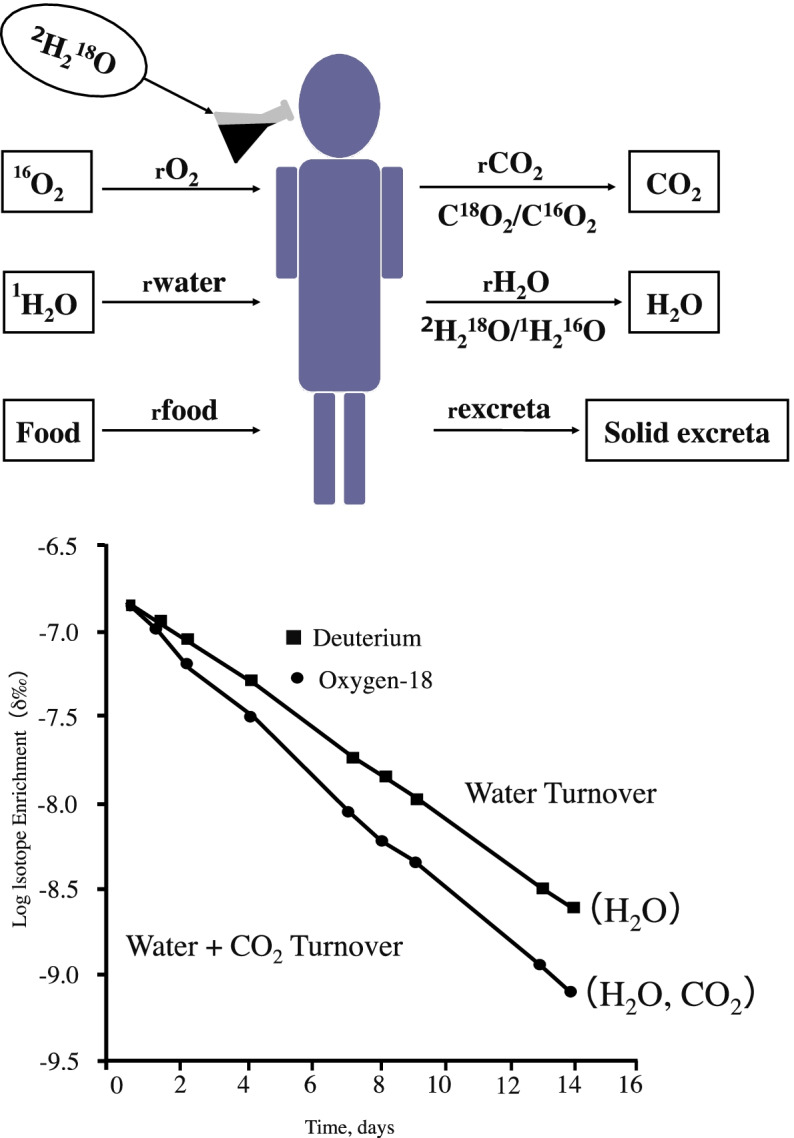
Basic concept for doubly labeled water (DLW) method

TEE was measured using the DLW method, as previously described, for 8 consecutive days. Figure 1 shows the basic theory of the DLW method. A known amount of water of known concentration (DLW, ^2^H_2_^18^O) is administered orally. The two isotopes equilibrate with TBW within a few hours and then are eliminated differentially from the body. ^18^O is eliminated from the body as water (H_2_^18^O) in urine, sweat, and body water vapor in exhaled breath. In addition, it is also eliminated as carbon dioxide (C^18^O_2_) in exhaled gas. However, ^2^H is eliminated from the body only as water (^2^H_2_O). The principle is that the resulting difference in the emission rates (or turnover rate) of the two isotopes can be used to determine the emission rate of carbon dioxide. After an overnight fast, baseline urine samples were collected from each participant, and DLW was administered orally. The dose of 0.05 g/kg estimated TBW of ^2^H (99.9 atom %) and 0.25 g/kg estimated TBW of ^18^O (10.0 atom %). The urine sample was collected at 3 h following administration (after 3-h equilibration) and on the morning of day 1, day 4, and day 8 after ingestion of DLW, which were used to measure the elimination rates of ^2^H and ^18^O. Each urine sample was collected and stored at − 80 °C until they were analyzed. All samples were analyzed using equipment installed at Nippon Sport Science University, Japan. The gas samples were analyzed by gas isotope ratio mass spectrometry (IRMS, IsoPrime, Micromass UK Ltd., UK). The gas samples for IRMS were prepared by equilibrating the urine sample with the particular gas to be analyzed. The ^2^H analysis was completed using standard hydrogen gas over a platinum catalyst. ^18^O analysis was performed using gas IRMS with carbon dioxide. These isotope ratio measurements were expressed as delta (δ) per mil (parts per 1000 or ‰). The isotopic ratios were then normalized against the international water standard, Vienna-Standard Mean Ocean Water (V-SMOW). The average standards for the analysis were 1.68 ‰ for ^2^H and 0.07 ‰ for ^18^O. TEE was expressed as the mean TEE per day during the study period. TEE was calculated using the following equations: TBW (L) = [(Dose × 99.8/20) (18.02/Ne (d^2^H)]/1.041 by the method of Schoeller et al. [15], where Dose is the dose of ^2^H_2_O, Ne is the ratio of ^2^H/^1^H in the standard, and d^2^H is the variance of the ^2^H enrichment before and after administration of isotopes for the participants. rCO_2_ (mol/d) = 0.4556 TBW (1.007 ko to 1.041 kh) was also determined by the Schoeller et al. method [15], where ko and kh are the elimination rates of ^18^O and ^2^H, respectively. TEE (kcal/day) = 22.4 [3.941 (rCO_2_/RQ) + 1.106 (rCO_2_)] was calculated by the Weir et al. method [16].

The statistical analysis program SPSS ver. 27 (IBM, Japan) was used for the statistical analysis. The results were presented as mean ± standard deviation (SD). We conducted a *t*-test for sex difference (Tables 1 and 2). Differences in the simple main effect between sex and age groups were analyzed by two-way ANOVA. Post hoc tests were not performed for comparisons between sex and age groups because of each two groups (Tables 3 and 4). Moreover, correlation and partial correlation analyses were used to determine the relationship between TEE and each anthropometric measurement (Table 5). Statistical differences were considered significant at *p* values less than 0.05. We structured and checked our paper using “STROBE Statement-Checklist of cross-sectional studies” [17].

**Table 1:** Comparison of physical characteristics with statistical data for sex differences analyzed by *t*-test

	Boys		Girls		*Sex*
	Mean	SD	Mean	SD	*p*
*n*	69		71		
Age, months	63.7	11.2	65.0	12.3	*0.509*
Height, cm	108.0	7.9	108.3	8.3	*0.816*
Weight, kg	18.4	3.9	18.3	3.3	*0.942*
Body mass index, kg/m^2^	15.6	1.7	15.5	1.2	*0.748*
Fat-free mass, kg	14.8	2.8	14.0	2.6	*0.069*
Fat mass, kg	3.6	1.7	4.4	1.3	0.003*
% Fat mass, %	19.1	5.2	23.7	5.1	< 0.001*
Fat-free mass index, kg/m^2^	12.6	1.1	11.8	0.8	< 0.001*
Fat mass index, kg/m^2^	3.0	1.1	3.7	1.0	< 0.001*

^*^Statistically different

**Table 2:** Comparison of absolute and relative total energy expenditure with statistical data for sex differences analyzed by *t*-test. Total daily energy expenditure estimated by doubly labeled water method

	Boys		Girls		*Sex*
	Mean	SD	Mean	SD	*p*
Total energy expenditure					
kcal/day	1535.7	488.8	1572.5	492.7	*0.658*
kcal/weight, kg/day	84.7	25.2	86.2	23.6	*0.715*
kcal/FFM, kg/day	105.0	31.8	112.9	29.9	*0.129*

**Table 3:** Comparison of physical characteristics with statistical data for sex- and age-related differences analyzed by two-way ANOVA

	Boys				Girls				*Difference*					
	3–4-year-old		5–6-year-old		3–4-year-old		5–6-year-old		*Sex*		*Age*		Sex*Age	
	Mean	SD	Mean	SD	Mean	SD	Mean	SD	*F*	*p*	*F*	*p*	*F*	*p*
*n*	24		45		20		51							
Age, month	51.4	6.6	70.3	6.6	48.5	7.7	71.5	6.1	*0.5*	*0.492*	*304.4*	< 0.001*	*2.9*	*0.091*
Height, cm	100.6	6.0	111.9	5.7	97.9	6.0	112.4	4.9	*1.2*	*0.267*	*163.2*	< 0.001*	*2.5*	*0.115*
Weight, kg	15.6	2.5	19.8	3.8	15.0	2.1	19.6	2.7	*0.6*	*0.449*	*64.2*	< 0.001*	*0.2*	*0.691*
Body mass index, kg/m^2^	15.4	15.7	15.7	1.8	15.6	1.0	15.5	1.3	*0.0*	*0.996*	*0.1*	*0.703*	*0.7*	*0.394*
Fat-free mass, kg	12.5	2.0	16.0	2.3	11.0	1.4	15.1	1.9	*10.7*	0.001*	*110.1*	< 0.001*	*0.7*	*0.405*
Fat mass, kg	3.2	0.9	3.8	2.0	4.0	1.0	4.5	1.4	*8.0*	0.005*	*4.2*	0.043*	*0.1*	*0.754*
% Fat mass, %	20.2	4.2	18.6	5.6	26.5	4.0	22.6	5.0	*33.0*	< 0.001*	*9.2*	0.003*	*1.6*	*0.204*
Fat-free mass index, kg/m^2^	12.3	1.2	12.7	1.0	11.4	0.6	11.9	0.8	*22.6*	< 0.001*	*7.9*	0.006*	*0.0*	*0.927*
Fat mass index, kg/m^2^	3.1	0.8	3.0	1.3	4.2	0.8	3.5	1.0	*18.1*	< 0.001*	*3.9*	*0.500*	*1.8*	*0.185*

^*^Statistically different

**Table 4:** Comparison of absolute and relative total energy expenditure with statistical data for sex- and age-related differences analyzed by two-way ANOVA

	Boys				Girls				*Difference*					
	3–4-year-old		5–6-year-old		3–4-year-old		5–6-year-old		*Sex*		*Age*		Sex*Age	
	Mean	SD	Mean	SD	Mean	SD	Mean	SD	*F*	*p*	*F*	*p*	*F*	*p*
Total energy expenditure														
kcal/day	1260.9	357.8	1682.3	489.0	1265.2	408.0	1693.1	473.3	*0.0*	*0.928*	*26.4*	< 0.001*	*0.0*	*0.969*
kcal/weight, kg/day	83.2	29.2	85.4	23.2	84.9	26.6	86.7	22.6	*0.1*	*0.745*	*0.2*	*0.651*	*0.0*	*0.957*
kcal/FFM, kg/day	104.4	36.8	105.3	29.1	115.8	37.0	111.8	26.9	*2.5*	*0.116*	*0.1*	*0.786*	*0.2*	*0.665*

^*^Statistically different

**Table 5:** Relationship between total energy expenditure, body size, and body composition based on correlation and partial correlation coefficients

Subject	*n*	Covariates	Height	Weight	FFM	FM	%fat
All	140	none	0.465**	0.473**	0.491**	0.243**	− 0.032
		Age	0.324***	0.342***	0.361***	0.189*	0.054
		FFM	0.068	0.063	-	0.063	0.080
		FFM and FM	0.058	0.000	-	-	0.077

^*^ p < *0.05*, ** p < *0.01*, *** p < *0.001*

## Results

Table 1 shows sex-wise physical characteristics and body composition of the participants. There were no differences between the two sexes in terms of physical characteristics such as age, height, and weight, but girls showed significant differences in FM, %FM, and FMI. Contrarily, FFMI values were significantly high in boys. Table 2 shows sex-wise absolute and relative TEE values. There was no difference observed between the two sexes. TEE was 1535.7 ± 448.8 and 1572.5 ± 492.7 kcal/day for boys and girls, respectively. TEE per body weight was 84.65 ± 25.23 and 86.16 ± 23.62 kcal/kg/day, respectively, for boys and girls; TEE per FFM was 104.96 ± 31.76 kcal/kg/day for boys and 112.92 ± 29.87 kcal/kg/day for girls.

Table 3 shows the physical characteristics and body composition of the participants shown in Table 1, segregated into age groups of 3–4- and 5–6-year-olds, and Table 4 shows the absolute and relative values of TEE by sex and age group. There was no interaction between sex and age group. Age group differences are shown for all parameters except BMI and FMI, and gender differences were significant for all body composition indices. The absolute values of TEE showed a significant age group difference, whereas the relative values showed no difference. TEE for boys and girls was 1260.9 ± 357.8 and 1265.2 ± 408.0 in the 3–4-year-old group and 1682.3 ± 489.0 and 1693.1 ± 473.3 kcal/day in the 5–6-year-old group, respectively. Contrarily, the relative values did not show any difference between the sexes or age groups. TEE per body weight were 83.17 ± 29.17 and 84.87 ± 26.26 in the 3–4-year-old group and 85.45 ± 23.17 and 86.67 ± 22.60 kcal/kg/day in the 5–6-year-old group for boys and girls, respectively. TEE per FFM were 104.37 ± 36.85 in the 3–4-year-old group and 105.28 ± 29.13 in the 5–6-year-old group for boys, and 115.80 ± 36.98 and 111.80 ± 26.93 kcal/kg/day for girls, respectively.

Table 5 shows the relationship between TEE, body size, and body composition. All parameters except %fat showed significant positive correlations with TEE, and FFM showed the highest correlation. After adjusting for age in months for FFM and FM, a significant relationship remained between TEE adjusted for age in months and each item. TEE was adjusted for FFM, and TEE was adjusted for both FFM and FM; however, no relationship was found with both the parameters.

Tables 6 and 7 show an international comparison of TEE using the DLW method in recent studies for boys and girls, respectively. TEE is presented in absolute and relative per body weight values, and subject information such as race is indicated. The results for this study are also presented for children aged 3–6 years (Table 2), 3–4 years, and 5–6 years (Table 4), respectively. The number of participants in this study was much larger than those seen in previous studies. The absolute and relative values for boys were comparable to those of other studies, while the absolute values for girls were comparable, but the relative values were slightly higher.

**Table 6:** International comparison of total energy expenditure estimated by doubly labeled water method in boys

**Boys**						Total energy expenditure						
Age (years)			*n*	Weight		kcal day^−1^		kcal kg^−1^ per day		Country	Race	Reference
Mean or range	SD	Range		Mean	SD	Mean	SD	Mean	SD			
**5.3**	**0.9**	**3.3 to 6.9**	**69**	**18.7**	**3.9**	**1535.7**	**488.8**	**84.7**	**25.2**	**JPN**	**Asian**	**This study**
**4.3**	**0.6**	**3.3 to 4.9**	**24**	**15.6**	**2.5**	**1260.9**	**357.8**	**83.2**	**29.2**	**JPN**	**Asian**	**This study**
**5.9**	**0.6**	**5.0 to 6.9**	**45**	**19.8**	**3.8**	**1682.3**	**489.0**	**85.4**	**23.2**	**JPN**	**Asian**	**This study**
2.5–3.4			15	15.0	1.7	1207	181	81.5	15.2	UK		[18]
3			13	15.5		1293	158	83.9	11.5	UK		[19]
3.5–4.4			16	16.9	2.3	1301	211	78.2	14.5	UK		[18]
4.2	0.9		14	17.6	1.3	1479	196	84.0	9.6	Chile		[20]
4.2–6.9			22	19.5	4.1	1396	272	71.6		USA		[21]
4.5	0.9	3 to 5	48	17.4	2.7	1197.0	192.0	69.4	8.1	USA	*1	[22]
5	1	4 to 6	16	20.3	4.3	1440.0	271.0	71.5	8.0	USA	Caucasian, *2	[23]
5			12	18.9		1644	179	87.5	10.0	UK		[19]
5.1	0.9	4 to 6	11	16.9	0.5 *a	1320	60 *a			JPN	Asian	[6]
5.1	1.1	3 to 6	51	19.9	0.4	1457	382			UK		[24]
5.1	0.8		8	19.1	3.4	1385	220	73.6	12.4	Guate		[25]
5.3	0.9		11	21.3	4.7	1575	303	73.9		USA		[26]
5.3	0.8		25	20.1	3.8	1382	255	68.8		US-Can		[27]
5.4	0.3		15	21.1	3.9	1415	252	67.1		USA		[28]
5.5	0.7	4 to7	12	24.2	7.2	1678.0	603.0				White	[29]
5.5	0.2		21	20.6	4.3	1498.0	172.0			Sweden		[30]
5.5	0.7		10	21.3	3.9	1505	125	70.7		US-Can		[27]
6.0		4.5 to 7	32	20.6		1641.0				UK		[31]
6.4	0.9		11	25.2	6.6	1804	614	71.6		USA		[26]

*1, White, Black, Hispanic, Asian, Multiracial subj

^*^2, the TEE/weight data (kcal/kg/day) was calculated by authors using the raw data described in previous studies

^*^a, standard error of the mean (SEM)

**Table 7:** International comparison of total energy expenditure estimated by doubly labeled water method in girls

**Girls**						Total energy expenditure						
Age (years)			*n*	Weight		kcal day^−1^		kcal kg^−1^ per day		Country	Race	Reference
Mean or range	SD	Range		Mean	SD	Mean	SD	Mean	SD			
**5.4**	**1.0**	**3.1 to 6.9**	**71**	**18.3**	**3.3**	**1572.5**	**492.7**	**86.2**	**23.6**	**JPN**	**Asian**	**This study**
**4.0**	**0.6**	**3.1 to 4.9**	**20**	**15.0**	**2.1**	**1265.2**	**408.0**	**84.9**	**26.6**	**JPN**	**Asian**	**This study**
**6.0**	**0.5**	**5.0 to 6.9**	**51**	**19.6**	**2.7**	**1693.1**	**473.3**	**86.7**	**22.6**	**JPN**	**Asian**	**This Study**
2.5–3.4			16	14.9	1.1	1125	211	75.8	15.0	UK		[18]
3			18	14.8		1138	136	77.7	10.3	UK		[19]
3.5–4.4			11	17.1	1.9	1264	238	74.2	11.0	UK		[18]
3.7	0.4	3 to 6	34	16.6	3.7	1290	334			UK		[24]
4.2–6.9			23	20.7	4.1	1346	296	65.0		USA		[21]
4.6	0.9	3 to 5	49	17.1	2.5	1122.0	140.0	66.3	6.8	USA	*1	[22]
4.6	0.9		14	17.6	1.8	1370	128	78.4	7.8	Chile		[20]
5	1	4 to 6	14	21.0	4.7	1309.0	304.0	62.0	6.2	USA	Caucasian, *2	[23]
5			16	18.5		1477	247	79.6	10.5	UK		[19]
5.1	0.9		26	20.1	3.5	1254	272	62.4		US-Can		[27]
5.1	0.9	4 to 6	11	18.0	0.5 *a	1282	57 *a			JPN	Asian	[6]
5.4	0.8		17	19.8	3.2	1383	207	69.8		US-Can		[27]
5.4	0.6		8	18.5	1.4	1247	262	67.4	12.9	Guatema		[25]
5.5	0.1		18	20.3	4.3	1373.0	131.0			Sweden		[30]
5.5	0.9		11	21.5	5.3	1365	330	63.5		USA		[26]
5.5	0.4		13	18.9	2.5	1347	184	71.3		USA		[28]
5.7		4.5 to 7	31	19.6		1433.0				UK		[31]
6.4	1.0	4 to7	12	26.4	6.4	1536.0	363.0				White	[29]
6.6	0.9		11	24.8	6.7	1815	392	73.2		USA		[26]

*1, White, Black, Hispanic, Asian, Multiracial subj

*2, the TEE/weight data (kcal/kg/day) was calculated by authors using the raw data described in previous studies

*a, standard error of the mean (SEM)

## Discussion

We have determined the energy consumption of pre-school and school-going Japanese children using the DLW method. The current Japanese dietary reference intake (Japanese-DRI) [32] is tentatively determined based on data from the DLW study of pre-school children in North America and European countries, which indicate that the EERs are 1300 for boys aged 3–5 years and 1250 kcal/day for girls [6]. There is limited data on TEE, calculated using the DLW method, for pre-school and school-going children including Japanese children. Only two studies have examined the TEE of 8 and 23 Japanese pre-school children, respectively [6, 33]. Therefore, the results of this study, which includes a larger number of participants, is important for establishing accurate estimated energy requirements (EER) of Japanese and Asian pre-school children. Comparisons with previous data for the same age in other countries are shown in Table 6 for boys and Table 7 for girls. The tables show the results from this study for all participants and age groups for boys and girls, respectively. In addition, most of the previous studies were from Western countries with only two studies based on Asians with pre-school and school-going Japanese children. The results of our study are comparable with or slightly higher than those reported in previous studies.

In addition, the Japanese-DRI [32] indicates that the reference height for 3–5-year-olds is 103.6 cm for boys and 103.2 cm for girls, and the reference weight is 16.5 kg for boys and 16.1 kg for girls. Children aged 5.2 years in a study by Nishimoto et al. [33], in comparison with those from two previous studies on Japanese participants, had a height of 96.2 cm and a weight of 13.4 kg, which were smaller than those according to the Japanese-DRI standards. Their TEE was also small at 1133 kcal/day. However, this research was a special study of healthy children of short stature (*n* = 8). In another study by Yamada et al. [6], children aged 5.1 years had a height of 107.2 cm and a weight of 17.5 kg, which were slightly larger than those according to the Japanese-DRI standards [32], but the TEE was the same at 1300 kcal. In the present study, the 3–4-year-old children were slightly small in height (100.6 for boys and 97 cm for girls) and weight (15.6 for boys and 15.0 kg for girls). Their TEE was 1260.9 for boys and 1265 kcal for girls, which was similar to the TEE recommendations of the Japanese-DRI [32]. Therefore, the results of this study are similar or slightly higher than those reported by Yamada et al. [6]. In terms of TEE per body weight, the previous study was in the 70 kcal/kg range, while the present study was in the 80 kcal/kg range for both boys and girls. Body weight was composed of body fat mass and lean mass (weight = FM + FFM). If Japanese children have a lower body FM, that is, a lower degree of obesity than that in Westerners, the value of TEE per body weight is likely to be higher. Comparing the frequency of obesity in Japanese and Western children, the results were clearly higher in Westerners [34]. Yamada et al. [6] and Komura et al. [10] reported that changes in TEE were strongly influenced by FFM in Japanese pre-school children or pre-teens, and FFM can be used to predict TEE. In this study, TEE and FFM were significantly correlated. It is also necessary to measure body composition in order to compare TEE per FFM, which is an issue that needs to be discussed in the future. In addition, this study did not examine activity energy expenditure (AEE). It is clear that AEE affects TEE throughout lifespan, especially at 5–10 years of age [11], and so the differences in TEE per FFM in this study compared to other countries may be due to differences in AEE. It was not possible to study this further with the data we currently have. Therefore, we believe that in the future, it is imperative to evaluate TEE in association with AEE using accelerometry or model with two components (activity and basal expenditure).

Next, we compared the TEE results obtained from the estimation equation with the results of this study. The TEE indicated by the DRI of Japanese children with a “normal” physical activity level (PAL) in 2020 data was 1300 for boys and 1250 kcal/day for girls in 3–5-year-old children and 1550 kcal/day for boys and 1450 kcal/day for girls in 6–7-year-old children, respectively [35]. Considering the age structure, the results of this study were slightly higher than those of the others (this study: 3–4-year-old boys; 1260.9 and girls; 1265 kcal/day, 5–6-year-old boys; 1686.2 and girls 1693.1 kcal/day). This tendency is consistent with the results of a previous study [10], which showed data for Japanese children between the ages of 10 and 12. In other words, it is possible that the DRI for Japanese children may be slightly underestimated. Contrarily, the TEE values calculated from the data of the participants in this study using the formula for estimating DRI (developed based on the TEE obtained by the DLW method in the USA and Canada) [36] were 1605.6 for boys and 1551.3 kcal/day for girls. These values were comparable to the results of this study (boys; 1535.7 and girls; 1572.5 kcal/day). The average difference between the TEE from the estimation formula and the TEE of this study was only 69.8 for boys and 23.3 kcal/day for boys and girls, respectively. Furthermore, when compared to TEE predicted using Torun’s equation in accordance with the FAO/WHO/UNU 2004 Expert Consultation, Torun’s data was 1310.7 for boys and 1371.3 kcal/day for girls, which clearly showed to be lesser than that observed in the results of this study [7, 35].

We would like to emphasize that this study was unique in that it included a larger number of children than those in previous studies and that the TEE was presented separately for each age group. However, this study had several limitations. Previous studies have shown that children’s physical activity varies with the season and that they are especially less active in the winter months [37–41]. This study included data measured in spring and winter in a single region. Seasonal differences and effects were not taken into account. Similarly, in particular, Japan has a long north–south axis and lifestyles differ greatly from region to region (urban, rural, and mountainous areas). There have been a study examining regional differences in physical activity in Japanese children [42], which have shown no regional differences in results. However, this study has not evaluated TEE or AEE. Thus, it is necessary to take regional influences into consideration. Furthermore, TEE was affected by the amount of daily activity. Yamada et al. [6] showed a correlation between TEE and step count in pre-school children. Physical activity and energy expenditure are expected to vary between holidays and weekdays [43–45]. The measurement error in the dietary intake assessment is based on household information, where dietary intake tends to fluctuate, especially on weekends [46]. Further research is needed to include seasonal and regional effects in Japan, as well as the day of measurement. Several studies have suggested that there may be racial/ethnic differences in energy metabolism and whole-body or organ-specific composition, even for the same body size [47–49].

Regular physical activity is an important component of childhood physical health and is associated with increased skeletal muscle mass and strength. Children with low levels of physical activity may experience health problems such as muscle loss in the future [50]. Thus, reduced PA in children may affect healthy growth and development. Furthermore, in recent years, the lifestyles of people around the world have significantly changed during the COVID-19 pandemic, and it has been reported that their changes have a significant impact on well-being and physical activity. Gilbert [51] indicated that there was an association between COVID-19 restrictions and reduced PA.

At this time, there are limited data available on Asian populations. Therefore, further studies on children in Asian countries are needed in addition to studies on Japanese children [6]. Taken together, with further research, we hope that the data from this study on energy expenditure in large participants will help establish the original reference values for Japanese children, which will be able to serve future health education in Japanese children. In addition, since there are few Japanese original source data, the Japanese-DRI is presented using data from overseas children, so DRI in Japanese children is likely to be underestimated. Accordingly, it is necessary to further update the TEE data using the DLW method, including this study, to contribute to the construction of DRI that are applicable in real life.

## Data Availability

The data sets acquired and analyzed in this study contain a great deal of personal information about the participants. Therefore, it will be disclosed at the discretion of the corresponding author on reasonable request.

## Abbreviations

DLW: Doubly labeled water
TEE: Total energy expenditure
BMI: Body mass index
TBW: Total body water
FFM: Fat-free mass
FM: Fat mass
FFMI: Fat-free mass index
FMI: Fat mass index
EER: Estimated energy requirement
PAL: Physical activity level
DRI: Dietary reference intake
AEE: Activity energy expenditure
